# Primary intradiploic meningioma in the pediatric age-group

**DOI:** 10.4103/1817-1745.66680

**Published:** 2010

**Authors:** Mahadevan Sambasivan, Kumar Padmanabhan Sanal, Sambasivan Mahesh

**Affiliations:** Department of Neurosurgery, Cosmopolitan Hospitals, Murinjapalam, Pattom P.O., Trivandrum, Kerala-695 004, India

**Keywords:** CT finding, histopathology, intradiploic menigioma, pediatric age-group, treatment strategy

## Abstract

The authors report a pediatric patient who presented with a slow-growing swelling on the scalp. Computed tomography (CT) of the head revealed an osteolytic intradiploic lesion of the cranial vault. The lesion was excised in toto, and histopathological examination revealed benign intradiploic meningioma. The possible etiology, clinical findings, CT appearance, differential diagnosis, and treatment strategy are discussed.

## Introduction

Approximately 1% of the meningiomas arise in extradural sites.[1,2] The meningiomas that originate in the skull have been referred to as calvarial, intraosseous, or intradiploic. Primary intradiploic meningiomas are relatively rare cranial lesions. Osteoblastic and osteolytic subtypes of intradipolic meningiomas have been described. Of these, the osteolytic subtype is most uncommon and is also more likely to be malignant than the osteoblastic type. Intradiploic meningiomas should be considered in the differential diagnosis of osteoblastic or osteolytic skull lesions.

## Case Report

A 13-year-old boy was admitted with the complaint of a swelling on the right side of the head near the temple since 6 months. The parents reported that the swelling had gradually increased in size over 2 months. There was no history of similar swellings on the head or elsewhere in the body. There was no history of pain, fever, headache, vomiting, seizures, or visual disturbances.

**Figure 1:**
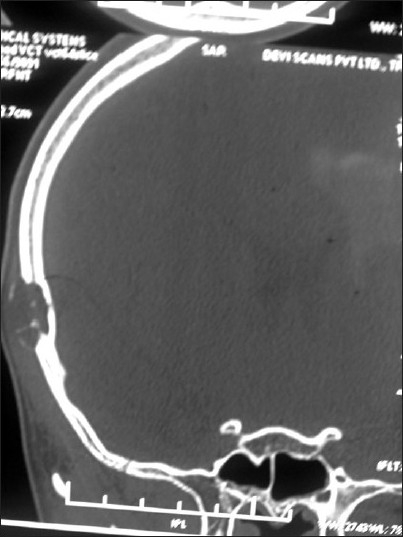
Plain CT head, coronal section, shows a focal osteolytic lesion in the right high temporal region, with interruption of the outer table and an intact inner table of the skull

**Figure 2:**
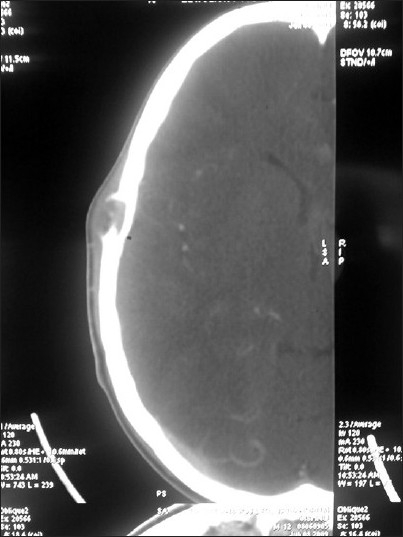
CT head, axial section, shows an osteolytic lesion in the right high temporal region, with interruption of the outer table and an intact inner table of the skull

**Figure 3:**
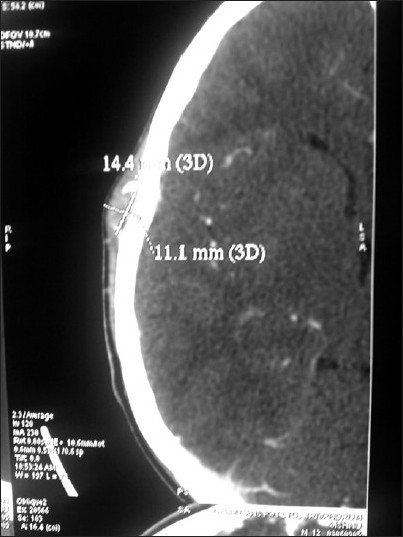
Contrast CT scan, axial section, shows homogeneous enhancement of the lesion located in the right high temporal region. Note the intact inner table and interruption of the outer table of the skull

General examination showed a moderately built and moderately nourished boy. He was not anemic, jaundiced, or cyanosed, and there was no peripheral lymphadenopathy. He had blood pressure of 110/70 mm Hg and a pulse rate of 76/min. No abnormality was detected either in the central nervous system or the cardiovascular system. The patient had an upper respiratory tract infection, which was controlled with antibiotics. Local examination revealed a small swelling of about 2 cm in diameter in the right upper temporal region. The skin over the swelling appeared normal. There was no visible pulsation or audible bruit. The surface of the lesion was smooth and the consistency was hard. The swelling appeared to arise from the bone. Plain CT of the head [Figures 1 & 2] showed a focal, lytic, punched-out lesion in the right temporal region, adjacent to the temporoparietal suture line. The inner table of the skull appeared intact but the outer table showed erosion. Contrast CT scan [Figure 3] showed contrast enhancement of the lesion. The clinical findings and the CT appearance were suggestive of either an aneurysmal bone cyst or eosinophilic granuloma. Under general anesthesia, the lesion was excised in toto, along with the overhanging bony ridges. The inner table of the skull was found to be intact. Histopathological examination revealed a neoplasm composed of spindle cells arranged in whorls. There were areas showing concentric whorling of the tumor cells. The tumor showed extensive myxoid degeneration. The appearances were suggestive of benign intradiploic meningioma.

The postoperative period was uneventful and patient was discharged on the 7^th^ postoperative day with advice to come for periodic follow-up.

## Discussion

It is likely that intradiploic meningiomas originate from arachnoid cells entrapped in the bone.[1,2] Head trauma, abnormal cranial moulding and embryogenesis, and arachnoid cells accompanying blood vessels and cranial nerves as they traverse the skull, can all result in the entrapment of arachnoid cells or meningocytes in the bone. It is presumed that intradiploic menigiomas arise from these entrapped cell rests in the calvarium.

The classification of primary extradural meningiomas was introduced by Lang and colleagues and is as follows:

- Type I: Tumors that are purely extracalvarial
- Type II: Tumors those are purely calvarial
- Type III: Calvarial tumor with extracalvarial extension

Based on their anatomical location, these types are further divided into convexity or skull-base tumors. Intradiploic meningiomas usually belong to either type 2 or type 3; the present case belongs to type 3. These tumors tend to occur in young patients but there is a second peak between the 5^th^ to 7^th^ decades of life. The present case is a 13-year-old boy who presented with a solitary lesion in the pterional area. Intradiploic meningiomas are usually solitary. Convexity intradiploic meningiomas most commonly present as slow-growing scalp masses, with possible relation to cranial suture. Common locations include the periorbital region and fronto, temporo parietal region.

Radiographically, intradiploic meningiomas are typically either osteoblastic or osteolytic.[1,2] Mixed versions also have been reported. The majority of these tumors cause hyperostosis, which may mimic fibrous dysplasia. Most of these tumors are benign but malignant transformation is also described. The osteolytic subtype of intradiploic meningiomas are more likely to be malignant than the osteoblastic subtype. Intradiploic meningiomas should be considered in the differential diagnosis of patients presenting with osteoblastic or osteolytic skull lesions. The osteolytic lesions typically cause thinning, expansion, and interruption of the inner or outer tables of the skull, and these lesions also enhance homogeneously after contrast administration. As of 2007, only 16 cases of the rare osteolytic subtype have been reported in the literature.[1–4] In the present case, the lesion appeared as an osteolytic variety, with interruption of the outer table and an intact inner table; it also showed contrast enhancement. The differential diagnosis of a solitary osteolytic skull lesion includes hemangioma, chondroma, chondrosarcoma, dermoid, myeloma, plasmacytoma, giant cell tumor, aneurysmal bone cyst, eosinophilic granuloma, metastatic deposit, and intradiploic menigioma. Biopsy and histopathological examination is necessary to confirm the diagnosis.

Wide surgical resection of intradiploic meningioma is the treatment of choice and is potentially curative if surgery is possible.[3,4] However, patients with tumors that cannot be completely resected and that are histologically benign and neurologically asymptomatic may be followed up using serial CT/MRI. Twenty-six percent of these tumors may show evidence of atypical or malignant changes. These patients may be considered for adjuvant therapy, which may include radiation or chemotherapy.

The present case belongs to the osteolytic subtype of intradiploic meningioma. The tumor was excised in toto and histopathological examination showed evidence of a benign type of intradiploic meningioma. The patient was advised to come for periodic follow-up to rule out recurrence of the lesion.
